# Novel postoperative nomograms for predicting individual prognoses of hepatitis B-related hepatocellular carcinoma with cirrhosis

**DOI:** 10.1186/s12893-022-01789-4

**Published:** 2022-09-13

**Authors:** Liangliang Xu, Fuzhen Dai, Peng Wang, Lian Li, Ming Zhang, Mingqing Xu

**Affiliations:** 1 Department of Liver Surgery, West China Hospital, Sichuan University, Chengdu, 610041 Sichuan China; 2 Department of General Surgery, The First People’s Hospital of Longquanyi District, Chengdu, 610041 Sichuan China

**Keywords:** Hepatocellular carcinoma, Cirrhosis, Hepatitis B virus, Prognosis, Nomogram

## Abstract

**Background:**

Liver cirrhosis is a well-known risk factor for carcinogenesis of hepatocellular carcinoma (HCC). The aim of the present study was to construct individual prognostic models for HCC with cirrhosis.

**Methods:**

The clinical differences between HCC patients with and without cirrhosis were compared using a large cohort of 1003 cases. The patients with cirrhosis were randomly divided into a training cohort and a validation cohort in a ratio of 2:1. Univariate and multivariate analyses were performed to reveal the independent risk factors for recurrence-free survival (RFS) and overall survival (OS) in HCC patients with cirrhosis. These factors were subsequently used to construct nomograms.

**Results:**

Multivariate analyses revealed that five clinical variables (hepatitis B e antigen (HBeAg) positivity, alpha-fetoprotein (AFP) level, tumour diameter, microvascular invasion (MVI), and satellite lesions) and seven variables (HBeAg positivity, AFP level, tumour diameter, MVI, satellite lesions, gamma-glutamyl transpeptidase level, and histological differentiation) were significantly associated with RFS and OS, respectively. The C-indices of the nomograms for RFS and OS were 0.739 (*P* < 0.001) and 0.789 (*P* < 0.001), respectively, in the training cohort, and 0.752 (*P* < 0.001) and 0.813 (*P* < 0.001), respectively, in the validation cohort. The C-indices of the nomograms were significantly higher than those of conventional staging systems (*P* < 0.001). The calibration plots showed optimal consistence between the nomogram-predicted and observed prognoses.

**Conclusions:**

The nomograms developed in the present study showed good performance in predicting the prognoses of HCC patients with hepatitis B virus-associated cirrhosis.

## Background

Hepatocellular carcinoma (HCC) is the most common type of liver cancer, and it exerts a huge medical burden globally [1, 2]. Approximately 782,000 new cases of HCC and 746,000 deaths caused by HCC are reported worldwide each year [1]. More than half of these new cases and deaths are recorded in China due to the high prevalence of hepatitis B virus (HBV) infection in the country [3–5]. HCC is the sixth most common malignancy and the third leading cause of cancer-related death worldwide [1, 2]. Owing to the lack of symptoms, and the resultant lack of regular surveillance, in the early stage of HCC, only 5–10% of HCC patients are candidates for curative resection according to the Barcelona Clinic Liver Cancer (BCLC) staging system [2, 6]. Unfortunately, approximately 70% of HCC patients show recurrence within 5 years after curative resection [7]. Therefore, development of new treatment strategies and regular postoperative surveillance are imperative for increasing the long-term survival of HCC patients.

Cirrhosis is a well-known risk factor for HCC. Approximately 70% of patients with HCC have a background of cirrhosis [2, 8]. The annual incidence of HCC associated with an established cirrhotic state ranges from 2.5 to 6.6% [9–11], which is 2.79- to 45.0-fold higher than the range for HCC arising from a non-cirrhotic state, irrespective of the aetiology of liver disease [12]. The carcinogenesis of HCC in patients with cirrhosis is often related to the sequential progression of regenerative nodules to dysplastic nodules to well-differentiated HCC [13]. There are multiple clinical differences between patients with and without cirrhosis. A recent study of patients from Japan and America revealed that HCC patients with cirrhosis are more likely to have a high HBV or hepatitis C virus (HCV) load, increased α-fetoprotein (AFP) level, poor liver function, low platelet density, good pathological differentiation, and unfavourable postoperative prognosis than HCC patients without cirrhosis [14]. Meanwhile, the annual probability of postoperative recurrence of HCC in patients with cirrhosis is approximately 6–15% higher than that in patients without cirrhosis [14]. Furthermore, patients with cirrhosis are more likely to show decompensated cirrhosis post-surgery, with symptoms including ascites, variceal bleeding, encephalopathy, and/or jaundice [15, 16]. In this context, prognostic predictive models are imperative for the designation and initiation of personalized surveillance strategies and adjuvant therapies for this subset of HCC patients. Unfortunately, such predictive models have not been established specifically for HCC patients with cirrhosis.

Nomograms are widely accepted by several investigators for the prediction of the outcomes of various diseases [17–19]. They are constructed based on the independent risk factors of special endpoints and show more accuracy than commonly used staging systems [20]. Thus, the aim of this study was to compare the clinical characteristics of HCC patients with and without cirrhosis using a large cohort of HCC patients without macrovascular invasion, and construct nomograms for predicting the individual recurrence-free survival (RFS) and overall survival (OS) of HCC patients with cirrhosis. In addition, we compared the accuracies of the nomograms with those of conventional staging systems.

## Methods

### Study population

Patients who underwent curative resection for HCC from June 2006 to March 2015 in West China Hospital, Sichuan University, were retrospectively screened and included in this study. The inclusion criteria were the follows: (1) aged 18 years or older; (2) pathologically diagnosed with HCC; (3) positive hepatitis B surface antigen (HBsAg) test result; (4) capable liver reserve function (Child–Pugh grade A or B); and (5) underwent curative hepatectomy as an initial treatment. The exclusion criteria were as follows: (1) presence of HCC alongside cholangiocarcinoma or other types of tumours; (2) presence of MVI; (3) pathologically confirmed lymph node metastasis; (4) died in the hospital or lost to follow-up within 3 months post-surgery; (5) and missing data on important clinical variables, such as tumour diameter, HBV-DNA load, and pathology results. Finally, a total of 1003 patients who met the inclusion criteria were included in this study. Written informed consent for use of patient data was obtained from all the included patients. This study was approved by the ethics committee of West China Hospital, Sichuan University.

### Baseline and clinical variables

The demographic characteristics and clinicopathologic variables of each patient were extracted from the digital healthcare system of West China Hospital, Sichuan University. Demographic characteristics included age, sex, underlying liver diseases, hypertension, and diabetes mellitus. Preoperative variables included Child–Pugh grade; coagulation function; HBV-DNA load; AFP level, portal hypertension; white blood cell (WBC), neutrophil (NEU), lymphocyte (LYM), and platelet (PLT) concentrations; and gamma-glutamyl transpeptidase (GGT) level. Imageology variables included tumour location/diameter/number and major vascular invasion, which were determined using three-phase-enhanced computed tomography (CT) or magnetic resonance imaging (MRI) scans. Intraoperative variables included resection type, blood loss, and transfusion. The histological diagnosis of each patient was made by two professional pathologists who were blinded to the clinical information and laboratory findings of the patients. The differentiation grade was determined according to the criteria of the Edmondson-Steiner classification [21]. MVI was defined as the presence of tumour emboli in small vessels in surgical samples, and was detected through microscopy [22]. Satellite lesions were defined as separate nodules less than 2 cm in diameter around the main tumour [23]. Fibrosis status was determined using the Ishak Fibrosis Scale (scores 0 to 6) [24]. Patients with an Ishak score of 5 (incomplete cirrhosis) or 6 (definite cirrhosis) were categorised into the cirrhosis group, whereas the remaining patients were classified into the non-cirrhosis group.

### Follow-up

A regular follow-up strategy was utilised for all patients after discharge from the hospital. Routine blood tests; measurement of serum AFP level, HBV-DNA load, and liver function; and abdominal ultrasonography, CT, or MRI scan were performed in the first postoperative month, at 3-month intervals for the next 3 postoperative years, and every 6 months subsequently. For patients with positive HBV-DNA results before surgery or during follow-up, antiviral therapy (entecavir or lamivudine) was administered immediately. If indicated, bone scan or positron emission tomography was performed to confirm distant metastasis. Tumour recurrence was determined after at least two radiological examinations show new lesions with the typical appearance of HCC in the remnant liver, extrahepatic tissues, or organs. After diagnosis of recurrence, optimum treatment, including radiofrequency ablation, rehepatectomy, salvage liver transplantation, transarterial chemoembolization (TACE), or administration of sorafenib, or best care support was performed. Recurrence-free survival (RFS) was defined as the interval between surgery and the first diagnosis of recurrence. Overall survival (OS) was defined as the interval between surgery and death or the last follow-up. The follow-up was censored in August 2018.

### Statistical analysis

All statistical analyses were performed using SPSS version 24.0 (IBM SPSS Inc, Chicago, IL) and R software version 3.5.0 (http://www.r-project.org/). The cut-off values of continuous blood test variables were determined using the normal reference values, meanwhile, the cut-off values of age, AFP and intraoperative blood loss were determined age-specific cut-off values. AFP level and intraoperative blood loss were determined according the information reported in previous medical studies [25–27]. Categorical variables are expressed as number or percentage and were compared using Pearson’s Chi-square test or Fisher’s exact test. Normally distributed continuous variables are expressed as mean (standard deviation, SD) and were analysed using Student’s *t* test or the Mann–Whitney U test. Univariate and multivariate analyses were performed using a Cox proportional hazard model. Significant variables in the univariate analysis were integrated into the multivariate analysis to identify the independent risk factors for RFS and OS.

To construct the nomograms, the patients with cirrhosis were randomly assigned into a training cohort or a validation cohort in a ratio of 2:1. The nomograms were generated using the rms package in R software based on the independent risk factors identified in the training cohort. The predictive accuracy of the nomogram was measured using Harrell’s concordance index (C-index). Bootstrapping with 1000 resamples was performed to reduce the biased estimates. A higher C-index value represents a more accurate predictive ability. The calibration curves were applied to illustrate the agreement between the nomogram-predicted and the observed probabilities of recurrence. For internal validation, the total points of each patient in the validation cohort were calculated using the established nomograms. Thereafter, the total points were treated as a new factor for calculating the C-index and depicting the calibration curve. If there was no significant difference in C-index and calibration between the training and validation cohorts, stable performance of the nomograms was considered. In addition, the predictive accuracies of the nomograms were compared with those of other conventional staging systems using the rcorrp.cens package [17]. All statistical tests were two-tailed, and a P value < 0.05 was considered statistically significant.

## Results

### The fibrosis statuses of all included patients

**Fig. 1:**
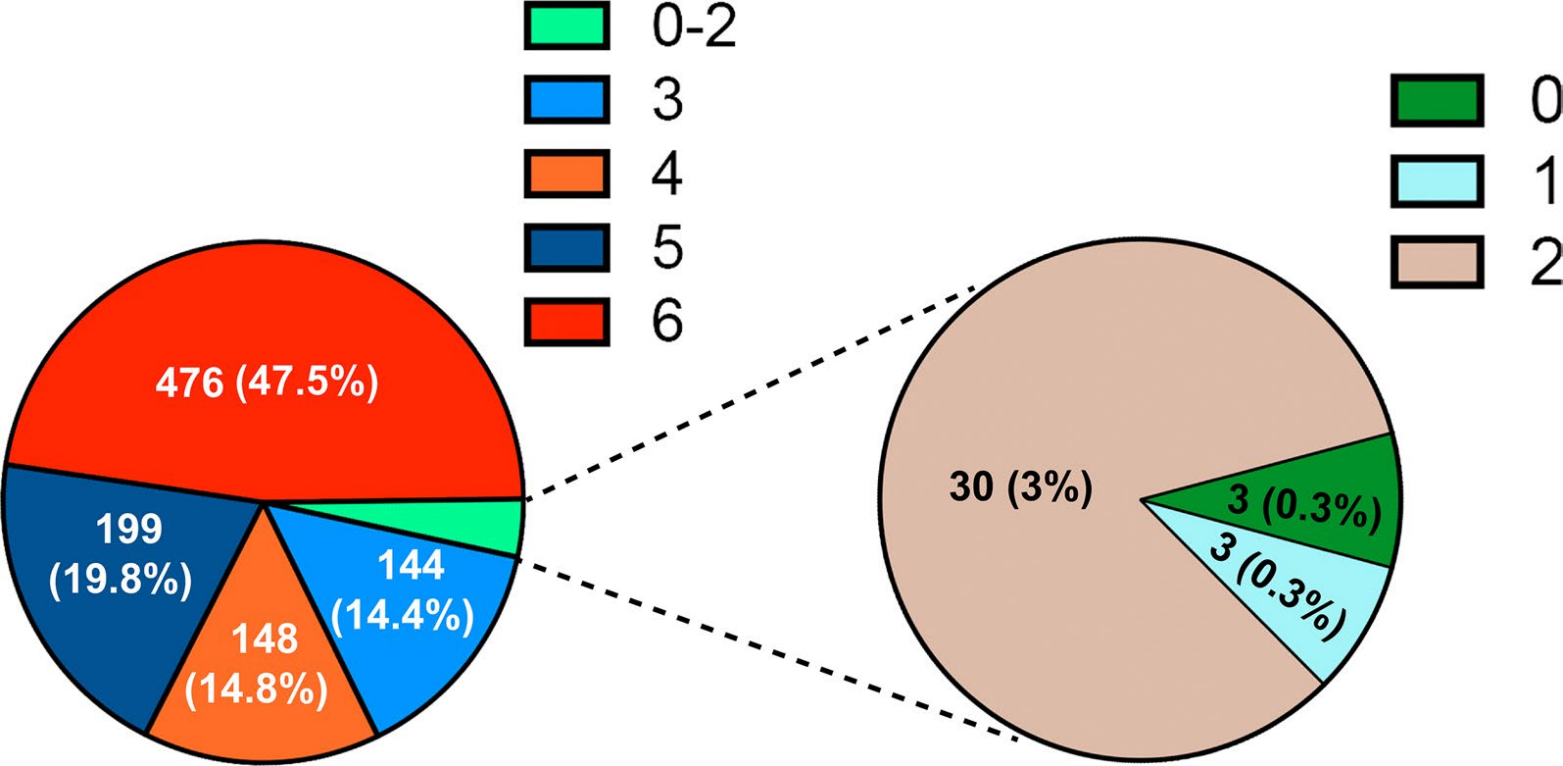
The fibrosis extent of included patients with HCC was evaluated by Ishak scoring system

A total of 1003 patients with HBV-related HCC who underwent curative liver resection between June 2006 and March 2015 were included in this study. The fibrosis status of each patient was evaluated using the Ishak staging system. As shown in Fig. 1, only 3 (0.3%) patients had a fibrosis score of 0, which means no fibrosis. Three (0.3%) patients had fibrosis score of 1, which indicates fibrous expansion of some portal areas, with or without a short fibrous septa. Thirty (3.0%) patients had a fibrosis score of 2, which indicates fibrous expansion of most portal areas, with or without a short fibrous septa. One hundred and forty four (14.4%) patients had fibrosis score of 3, which means fibrous expansion of most portal areas with occasional portal-to-portal bridging. One hundred and forty eight (14.8%) patients had a fibrosis score of 4, which denotes fibrous expansion of most portal areas with marked bridging (both portal-to-portal and portal-to-central). One hundred and ninety nine (19.8%) patients had a fibrosis score of 5, which means incomplete cirrhosis characterised by marked bridging and occasional nodules. The remaining 476 (47.5%) patients had a fibrosis score of 6, which means probable or definite cirrhosis. We classified patients with an Ishak score of 5 or 6 into the cirrhosis group (675, 67.3%), and the rest of the patients into the non-cirrhosis group (328, 32.7%).

### Clinical differences between the cirrhosis and non-cirrhosis groups

The clinical characteristics of the patients in the cirrhosis and non-cirrhosis groups are listed in Table 1. The percentage of patients in the cirrhosis group with a high HBV-DNA load; HBeAg positivity; elevated AFP level; decreased WBC, NEU, and PLT concentrations; prolonged prothrombin time (PT); increased international normalized ratio; decreased fibrinogen; and elevated total bilirubin was higher than that in the non-cirrhosis group. However, the percentage of patients in the cirrhosis group who were older than 60 years was lower than that in the non-cirrhosis group. Regarding tumour characteristics, the mean tumour diameter in the cirrhosis group was significantly smaller than that in the non-cirrhosis group. However, the percentage of patients in the cirrhosis group who had multiple tumour nodules was higher than that in the non-cirrhosis group. The percentage of patients in the cirrhosis group who underwent anatomical liver resection was 42.5%, which is significantly lower than the 56.9% recorded in the non-cirrhosis group. There were no other significant clinical differences between the cirrhosis and non-cirrhosis groups.

**Table 1:** Clinical differences between cirrhotic and non-cirrhotic patients with HCC

Clinical parameters	Non-cirrhosis (n = 328, %)	Cirrhosis (n = 675, %)	P-value	Clinical parameters	Non-cirrhosis (n = 328, %)	Cirrhosis (n = 675, %)	P-value
*Gender*			0.778	*ALT (IU/L)*			0.659
Male	275 (83.8)	570 (84.5)		≥ 50	112 (34.1)	240 (35.5)	
Female	53 (16.2)	105 (15.5)		< 50	216 (65.9)	435 (64.5)	
*Age (years)*			**0.014**	*AST (IU/L)*			0.385
≥ 60	101 (30.9)	152 (22.5)		≥ 40	153 (46.6)	334 (49.5)	
< 60	227 (69.1)	523 (77.5)		< 40	175 (53.4)	341 (50.5)	
*HBV-DNA (copies/mL)*			**0.002**	*ALB (g/L)*			0.054
≥ 10^3^	169 (51.4)	397 (58.8)		≥ 40	224 (68.2)	419 (62.1)	
< 10^3^	159 (48.6)	278 (41.2)		< 40	104 (31.8)	256 (37.9)	
*HBeAg*			**0.002**	*GGT (IU/L)*			0.363
Positive	43 (13.1)	149 (22.1)		≥ 60	170 (51.7)	329 (48.7)	
Negative	285 (86.9)	526 (77.9)		< 60	158 (48.3)	346 (51.3)	
*AFP (ng/mL)*			**0.032**	*Child–pugh*			0.961
≤ 20	116 (35.3)	227 (33.6)		A	323 (98.6)	666 (98.6)	
20–400	65 (19.7)	182 (27.0)		B	5 (1.4)	9 (1.4)	
≥ 400	147 (45.0)	266 (39.4)		Tumor diameter, mean (SD), cm	7.44 (3.91)	5.53 (3.25)	**< 0.001**
*WBC (× 10*^*9*^*/L)*			**< 0.001**	*Tumor number*			**0.015**
≥ 4	297 (90.6)	502 (74.3)		Multi	36 (11.1)	113 (16.8)	
< 4	31 (9.4)	173 (25.7)		Single	292 (88.9)	562 (83.2)	
*NEU (× 10*^*9*^*/L)*			**< 0.001**	*Differentiation*			0.628
≥ 3.56	184 (56.0)	239 (35.4)		I + II	193 (58.8)	383 (56.8)	
< 3.56	144 (44.0)	436 (64.6)		III + IV	135 (41.2)	292 (43.2)	
*LYM (× 10*^*9*^*/L)*			0.089	*MVI*			0.24
≥ 1.1	261 (79.5)	505 (74.8)		Present	128 (38.9)	238 (35.2)	
< 1.1	67 (20.5)	238 (35.2)		Absent	200 (61.1)	437 (64.8)	
*PLT (× 10*^*9*^*/L)*			**< 0.001**	*Satellite lesion*			0.328
≥ 100	275 (83.8)	409 (60.6)		Present	48 (14.5)	83 (12.3)	
< 100	53 (16.2)	266 (39.4)		Absent	280 (85.5)	592 (87.7)	
*PT (s)*			**< 0.001**	*Resection*			**< 0.001**
≥ 12.8	48 (14.5)	186 (27.5)		Anatomic	187 (56.9)	287 (42.5)	
< 12.8	280 (85.5)	489 (72.5)		Non-anatomic	141 (43.1)	388 (57.5)	
*INR*			**< 0.001**	*Blood loss (mL)*			0.872
≥ 1.15	47 (14.2)	172 (25.5)		≥ 400	272 (82.9)	567 (84.0)	
< 1.15	281 (85.8)	503 (74.5)		< 400	56 (17.1)	108 (16.0)	
*Fib (g/L)*			**< 0.001**	*Transfusion*			0.734
≥ 2	297 (90.6)	538 (79.7)		Yes	51 (15.6)	109 (16.1)	
< 2	31 (9.4)	137 (20.3)		No	277 (84.4)	566 (83.9)	
*TBIL (μmol/L)*			**0.02**				
≥ 28	8 (2.3)	36 (5.4)					
< 28	320 (97.7)	639 (94.6)					

Bold numbers indicate statistical significance

HBV-DNA, hepatitis B virus deoxyribonucleic acid; HBeAg, hepatitis B e antigen; AFP, alpha-fetoprotein; WBC, white blood cell; NEU, neutrophil; LYM, lymphocyte; PLT, platelet; PT, prothrombin time; s, second; INR, international normalized ratio; Fib, fibrinogen; TBIL, total bilirubin; ALT, alanine transaminase; AST, aspartate aminotransferase; ALB, albumin; GGT, gamma-glutamyl transpeptidase; SD, standard deviation; MVI, microvascular invasion

### Follow-up results and independent prognostic factors for recurrence-free survival and overall survival in HCC patients with cirrhosis

The median follow-up duration and median RFS for the cirrhosis group was 39 months (range, 1–120 months) and 25 months (range, 1–116 months), respectively. A total of 429 out of 675 patients in the cirrhosis group showed recurrence during follow-up. Among them, 80 (18.6%) patients underwent repeat liver resection, 10 (2.3%) underwent salvage liver transplantation, 65 (15.2%) underwent radio frequency ablation, 181 (42.2%) underwent TACE, 5 (1.2%) received sorafenib, and the remaining (20.5%) received best care support. The postoperative 1, 3, and 5-year RFS and OS rates in the cirrhosis group were 64.42%, 43.61%, and 31.97% and 88.79%, 63.15%, and 50.90%, respectively. Regarding the non-cirrhosis group, 217 (66.16%) patients showed postoperative recurrence. The postoperative 1, 3, and 5-year RFS and OS rates in the non-cirrhosis group were 62.25%, 41.10%, and 31.47% and 84.73%, 57.16%, and 46.42%, respectively.

The HCC patients with cirrhosis were randomly divided into a training cohort (n = 450) and a validation cohort (n = 225) in a 2:1 ratio. The training and validation cohorts were used to construct and validate the prognostic models, respectively. As shown in Table 2, except for aspartate aminotransferase (AST) level and the presence of MVI, there were no other significant differences between the cohorts. As listed in Table 3, univariate analysis of the training cohort revealed that HBV-DNA, HBeAg, AFP level, NEU concentration, LYM concentration, GGT level, tumour diameter, number of tumours, histologic differentiation, MVI, and satellite lesions were significantly associated with RFS. The parameters significantly associated with OS were HBV-DNA, HBeAg, AFP level, NEU concentration, LYM concentration, PLT concentration, AST level, GGT level, tumour diameter, histologic differentiation, MVI, satellite lesions, and type of hepatectomy. These variables were subsequently included in the multivariate analyses. The results showed that HBeAg positivity (HR, 1.426; 95% confidence interval (CI) 1.085–1.874; *P* = 0.011), elevated AFP level (HR, 1.576; 95% CI 1.237–2.008; *P* < 0.001), large tumour diameter (HR, 1.358; 95% CI 1.060–1.739; *P* = 0.015), presence of MVI (HR, 1.943; 95% CI 1.527–2.471; *P* < 0.001), and presence of satellite lesions (HR, 1.794; 95% CI 1.305–2.467; *P* < 0.001) were the independent risk factors for RFS, whereas HBeAg positivity (HR, 1.517; 95% CI 1.105–2.081; *P* = 0.01), elevated AFP level (HR, 1.477; 95% CI 1.113–1.960; *P* < 0.001), large tumour diameter (HR, 1.383; 95% CI 1.024–1.868; *P* = 0.035), presence of MVI (HR, 2.113; 95% CI 1.594–2.802; *P* < 0.001), presence of satellite lesions (HR, 1.509; 95% CI 1.050–2.167; *P* = 0.026), increased GGT level (HR, 1.416; 95% CI 1.051–1.907; *P* = 0.022), and poor histologic differentiation (HR, 1.411; 95% CI 1.078–1.847; *P* = 0.012) were the independent risk factors for OS.

**Table 2:** The baseline characteristics of cirrhotic patients with HCC in training and validation cohort

Clinical parameters	Training cohort (n = 450, %)	Validation cohort (n = 225, %)	P-value
Gender, male	374 (86.2)	176 (81.1)	0.092
Age, ≥ 60 y	96 (22.2)	50 (23.0)	0.214
HBV-DNA, ≥ 10^3^ copies/mL	246 (56.8)	137 (63.0)	0.367
HBeAg, positive	95 (21.9)	49 (22.6)	0.366
AFP, ≤ 20 ng/mL	173 (40.0)	83 (38.4)	0.866
AFP, 20–400 ng/mL	146 (33.7)	72 (33.3)	
AFP, ≥ 400 ng/mL	114 (26.3)	61 (28.2)	
WBC, ≥ 4 × 10^9^/L	174 (40.0)	84 (38.5)	0.498
NEU, ≥ 3.56 × 10^9^/L	156 (36.0)	74 (34.3)	0.657
LYM, ≥ 1.1 × 10^9^/L	327 (75.3)	160 (73.6)	0.632
PLT, ≥ 100 × 10^9^/L	266 (61.3)	132 (60.8)	0.909
PT, ≥ 12.8 s	125 (28.7)	55 (25.1)	0.388
INR, ≥ 1.15	115 (26.5)	51 (23.5)	0.408
Fib, ≥ 2 g/L	346 (79.6)	173 (79.9)	0.942
TB, ≥ 28 μmol/L	28 (6.5)	7 (3.2)	0.084
ALT, ≥ 50 IU/L	168 (38.7)	63 (29.0)	**0.015**
AST, ≥ 40 IU/L	229 (52.8)	93 (42.9)	0.017
ALB, ≥ 40 g/L	274 (63.1)	130 (59.9)	0.424
GGT, ≥ 60 IU/L	216 (49.8)	101 (46.5)	0.438
Child–pugh, Stage B	8 (1.8)	1 (0.5)	0.285
Tumor diameter, mean (SD), cm	5.65 (3.35)	5.28 (3.03)	0.178
Tumor number, multiple	73 (16.9)	36 (16.6)	0.931
Differentiation, III + IV	188 (43.4)	93 (42.9)	0.771
MVI, present	166 (38.2)	63 (29.0)	**0.02**
Satellite, present	56 (12.9)	24 (11.1)	0.493
Type of hepatectomy, anatomic	185 (42.7)	92 (42.3)	0.916
Blood loss, ≥ 400 mL	337 (86.8)	163 (75.0)	0.329
Transfusion, yes	67 (15.4)	38 (17.5)	0.523

Bold numbers indicate statistical significance

HBV-DNA, hepatitis B virus deoxyribonucleic acid; HBeAg, hepatitis B e antigen; AFP, alpha-fetoprotein; WBC, white blood cell; NEU, neutrophil; LYM, lymphocyte; PLT, platelet; PT, prothrombin time; s, second; INR, international normalized ratio; Fib, fibrinogen; TBIL, total bilirubin; ALT, alanine transaminase; AST, aspartate aminotransferase; ALB, albumin; GGT, gamma-glutamyl transpeptidase; SD, standard deviation; MVI, microvascular invasion

**Table 3:** Independent risk factors for RFS and OS verified by univariate and multivariate analyses

Clinical parameters	RFS		OS	
	HR (95% CI)	P-value	HR (95% CI)	P-value
*Univariate analysis*				
SEX (male/female)	0.894 (0.596–1.210)	0.365	0.816 (0.537–1.238)	0.338
Age (≥ 60/< 60 years)	0.797 (0.600–1.06)	0.118	0.844 (0.608–1.170)	0.308
HBV-DNA (≥ 10^3^/< 10^3^ copies/mL)	1.433 (1.112–1.845)	**0.005**	1.566 (1.159–2.117)	**0.004**
HBeAg (positive/negative)	1.397 (1.067–1.830)	**0.015**	1.487 (1.094–2.021)	**0.011**
AFP (≤ 20/20–400/≥ 400 ng/mL)	1.801 (1.427–2.273)	**< 0.001**	1.912 (1.463–2.497)	**< 0.001**
WBC (≥ 4/< 4 × 10^9^/L)	1.353 (0.957–1.913)	0.087	1.340 (0.900–1.995)	0.149
NEU (≥ 3.56/< 3.56 × 10^9^/L)	1.310 (1.033–1.662)	**0.026**	1.529 (1.165–2.007)	**0.002**
LYM (≥ 1.1/< 1.1 × 10^9^/L)	1.331 (1.005–1.763)	**0.046**	1.430 (1.027–1.992)	**0.034**
PLT (≥ 100/< 100 × 10^9^/L)	1.205 (0.949–1.530)	0.126	1.424 (1.075–1.886)	**0.014**
PT (≥ 12.8/< 12.8 s)	0.953 (0.720–1.260)	0.735	0.929 (0.673–1.283)	0.656
INR (≥ 1.15/< 1.15)	0.897 (0.688–1.169)	0.421	0.923 (0.678–1.257)	0.611
Fib (≥ 2/< 2 g/L)	1.279 (0.930–1.761)	0.131	1.336 (0.904–1.997)	0.147
TB (≥ 28/< 28 μmol/L)	0.740 (0.447–1.226)	0.243	0.690 (0.376–1.265)	0.23
ALT (≥ 50/< 50 IU/L)	1.154 (0.912–1.458)	0.233	1.106 (0.843–1.452)	0.467
AST (≥ 40/< 40 IU/L)	1.255 (0.995–1.583)	0.055	1.391 (1.061–1.823)	**0.017**
ALB (≥ 40/< 40 g/L)	0.899 (0.708–1.141)	0.382	0.781 (0.594–1.026)	0.075
GGT (≥ 60/< 60 IU/L)	1.540 (1.220–1.943)	**< 0.001**	2.027 (1.539–2.669)	**< 0.001**
Child–pugh (A/B)	1.489 (0.663–3.344)	0.335	0.978 (0.367–2.655)	0.979
Tumor diameter	1.734 (1.370–2.195)	**< 0.001**	1.960 (1.487–2.583)	**< 0.001**
Tumor number (multiple/solitary)	1.543 (1.157–2.057)	**0.003**	1.217 (0.863–1.716)	0.263
Differentiation (III + IV/I + II)	1.300 (1.035–1.634)	**0.024**	1.653 (1.269–2.152)	**< 0.001**
MVI (present/negative)	2.130 (1.689–2.687)	**< 0.001**	2.529 (1.934–3.307)	**< 0.001**
Satellite (present/negative)	1.906 (1.396–2.603)	**< 0.001**	1.743 (1.217–2.496)	**0.002**
Anatomic hepatectomy (yes/no)	1.214 (0.961–1.534)	0.103	1.432 (1.095–1.873)	**0.009**
Blood loss (≥ 400/< 400 mL)	2.846 (0.860–9.415)	0.087	3.861 (0.902–16.529)	0.069
Transfusion (yes/no)	1.655 (0.819–3.346)	0.16	1.351 (0.600–3.043)	0.468
*Multivariate analysis*				
HBeAg	1.423 (1.085–1.865)	**0.011**	1.528 (1.117–2.090)	**0.008**
AFP	1.546 (1.209–1.975)	**0.001**	1.417 (1.067–1.882)	**0.016**
Tumor diameter	1.038 (1.002–1.075)	**0.041**	1.054 (1.012–1.098)	**0.012**
MVI	1.906 (1.490–2.438)	**< 0.001**	2.009 (1.507–2.677)	**< 0.001**
Satellite lesion	1.791 (1.307–2.452)	**< 0.001**	1.458 (1.015–2.093)	**0.041**
GGT	ND	ND	1.388 (1.025–1.879)	**0.034**
Differentiation	ND	ND	1.375 (1.044–1.810)	**0.012**

Bold numbers indicate statistical significance

RFS, recurrence-free survival; OS, overall survival; HR, hazard ratio; CI, confidence interval; HBV-DNA, hepatitis B virus deoxyribonucleic acid; HBeAg, hepatitis B e antigen; AFP, alpha-fetoprotein; WBC, white blood cell; NEU, neutrophil; LYM, lymphocyte; PLT, platelet; PT, prothrombin time; s, second; INR, international normalized ratio; Fib, fibrinogen; TBIL, total bilirubin; ALT, alanine transaminase; AST, aspartate aminotransferase; ALB, albumin; GGT, gamma-glutamyl transpeptidase; SD, standard deviation; MVI, microvascular invasion; ND, no data

### Construction and validation of prognostic nomograms for recurrence-free survival and overall survival in HCC patients with cirrhosis

**Fig. 2:**
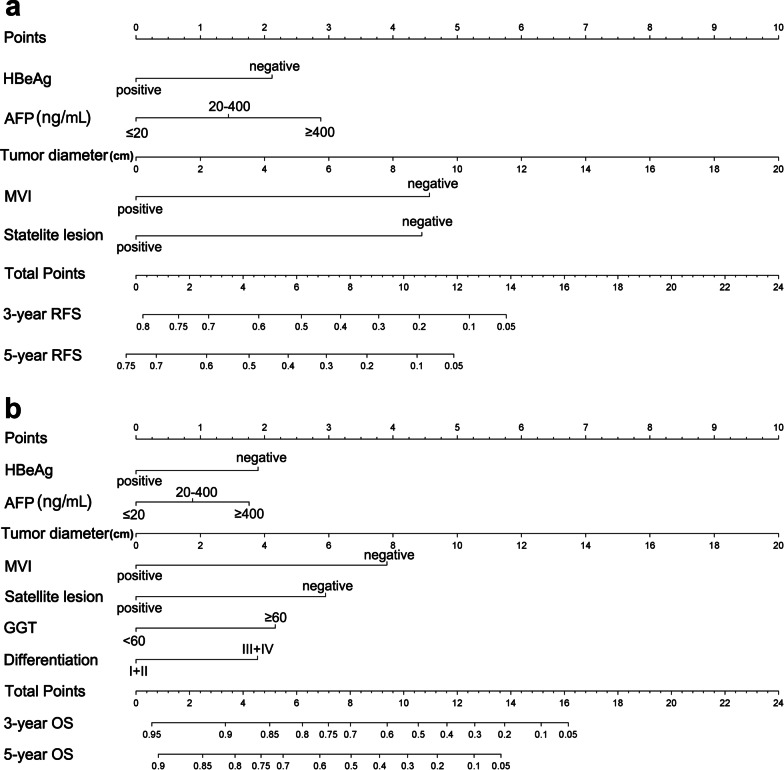
Nomograms for predicting recurrence-free survival (RFS) (**a**) and overall survival (OS) (**b**) in cirrhotic patients with HCC who underwent curative liver resection. *HBeAg* hepatitis B e antigen, *AFP* alpha-fetoprotein, *MVI* microvascular invasion, *GGT* gamma-glutamyl transpeptidase

**Fig. 3:**
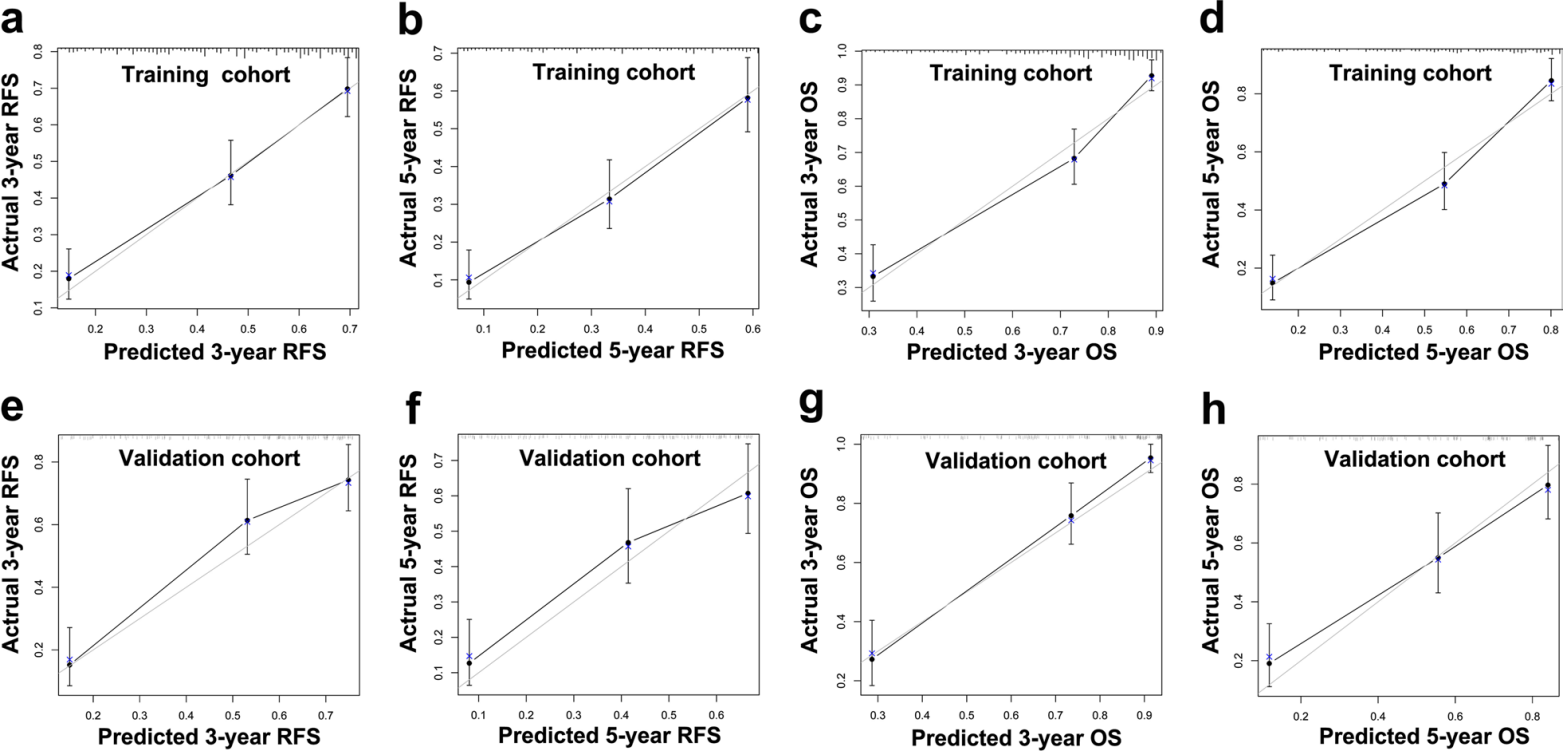
Calibration curves for predicting Recurrence-free survival (RFS) and overall survival (OS) using the nomograms. **a** and **b** 3 and 5-year RFS in the training cohort. **c** and **d** 3 and 5-year OS in the training cohort. **e** and **f** 3 and 5-year RFS in the validation cohort. **g** and **h** 3 and 5-year OS in the validation cohort

The independent risk factors outlined in the previous section were used to construct nomograms for RFS and OS in HCC patients with cirrhosis (Fig. 2). The nomograms showed promising accuracy in predicting prognoses. The bootstrap-corrected C-indices for the prediction of RFS and OS in the training cohort were 0.739 (95% CI 0.709–0.769; *P* < 0.001) and 0.789 (95% CI 0.759–0.819; *P* < 0.001), respectively. The calibration plots for the training cohort showed optimal consistency between the nomogram-predicted and actual observed 3- and 5-year RFS (Fig. 3a and b) and OS (Fig. 3c and d). For clinical use of these nomograms, the projection on the point scale indicates the score of each variable, and the total points are calculated by summing the scores of all variables. The projections of the total points on the prognostic scales represent the individual probability for 3- and 5-year RFS or OS.

For internal validation of the nomograms, the total points for each patient in the validation cohort were calculated using the nomograms. Thereafter, the total points were treated as a new factor used to calculate the C-indices and depict the calibration curves of RFS and OS, respectively. The results showed the C-indices for the prediction of RFS and OS in the validation cohort were 0.752 (95% CI 0.712–0.792; *P* < 0.001) and 0.813 (95% CI 0.775–0.851; *P* < 0.001), respectively, which are comparable to the C-indices for the prediction of RFS and OS in the training cohort. The calibration plots of the validation cohort also showed good agreement between the nomogram-predicted and actual observed 3- and 5-year RFS (Fig. 3e and f) and OS (Fig. 3g and h). These results indicated that the nomograms developed in the present study show a promising performance in predicting the prognoses of HCC patients with cirrhosis.

### Comparison of the predictive accuracies of the nomograms and conventional staging systems

To compare the accuracies of our nomograms with those of conventional staging systems in predicting the prognoses of HCC patients with cirrhosis, four routinely used staging systems (BCLC, the eighth version of American Joint Committee on Cancer (AJCC^8th^) staging manual, the Japan Integrated Staging Score (JIS score), and the Hong Kong Liver Cancer prognostic classification scheme (HKLC)) were selected and the discriminatory capacity of each prognostic system was compared using Harrel’s C-index (Table 4). In the training cohort, the C-index of the nomogram for RFS was 0.739, which is significantly higher (P < 0.001) than the C-indices of the BCLC, AJCC^8th^, JIS score, and HKLC systems. The C-index of the nomogram for OS was 0.789, which is also significantly higher (P < 0.001) than the C-indices of the BCLC, AJCC^8th^, JIS score, and HKLC systems. Similar results were also observed in the validation cohort. These data suggest that our nomograms are more feasible than conventional staging systems for predicting the prognoses of HCC patients with cirrhosis.

**Table 4:** Comparison of accuracy between present nomograms and four conventional staging systems via C-index

Prognostic system	RFS				OS			
	C-index	95% CI	P1^*^	P2^#^	C-index	95% CI	P1^*^	P2^#^
*Training cohort*								
Present nomogram	0.739	0.709–0.769	< 0.001	1.0 (Ref.)	0.789	0.759–0.819	< 0.001	1.0 (Ref.)
BCLC	0.615	0.587–0.643	< 0.001	< 0.001	0.633	0.601–0.665	< 0.001	< 0.001
AJCC^8th^	0.547	0.522–0.572	< 0.001	< 0.001	0.541	0.513–0.569	0.006	< 0.001
JIS score	0.541	0.517–0.566	0.001	< 0.001	0.524	0.497–0.551	0.09	< 0.001
HKLC	0.595	0.566–0.624	< 0.001	< 0.001	0.612	0.578–0.646	< 0.001	< 0.001
*Validation cohort*								
Present nomogram	0.752	0.712–0.792	< 0.001	1.0 (Ref.)	0.813	0.775–0.851	< 0.001	1.0 (Ref.)
BCLC	0.615	0.583–0.647	< 0.001	< 0.001	0.636	0.589–0.683	< 0.001	< 0.001
AJCC^8th^	0.562	0.527–0.597	< 0.001	< 0.001	0.576	0.535–0.617	< 0.001	< 0.001
JIS score	0.554	0.521–0.587	0.002	< 0.001	0.565	0.524–0.606	0.002	< 0.001
HKLC	0.601	0.559–0.643	< 0.001	< 0.001	0.616	0.567–0.665	< 0.001	< 0.001

Ref. reference; RFS, recurrence-free survival; OS, overall survival; CI, confidence interval; BCLC, Barcelona Clinic Liver Cancer staging system; AJCC^8th^, the eighth version of American Joint Committee on Cancer; JIS score, the Japan Integrated Staging Score; HKLC, the Hong Kong Liver Cancer prognostic classification scheme

^*^Represent significance of staging systems;

^#^Represent significance between present nomograms and conventional staging systems;

## Discussion

In this study, the fibrosis status of each included patient was evaluated according to the Ishak staging system. The Ishak staging system is a modified version of the Knodell system and includes seven stages of fibrosis (0–6), which are effective for distinguishing fibrosis status and architectural remodelling [24]. The Ishak staging system has been used in several recent clinical trials and is recommended as a tool in the Grading of Chronic Virus Hepatitis by the World Health Organization [28, 29]. The proportion of patients with cirrhosis in our cohort was 67.3%, which was in line with the percentages reported in previous studies [2, 8]. To the best of our knowledge, the present study is the first study in which the clinical characteristics of HCC patients with and without cirrhosis were compared using a large sample size of patients with HBV-related HCC. The results showed that the HCC patients in the cirrhosis group were more likely to be younger and have an elevated AFP level; decreased WBC, NEU, and PLT concentrations; poor coagulation function; smaller tumour size; and multiple tumours. Some of these findings are in line with the results of a recent study of Japanese and American patients with background aetiologies of cirrhosis including HBV infection (32.5%), HCV infection (58.7%), alcohol abuse (3.7%), and others (5.1%) [14]. These evidence indicate that patients with cirrhosis have a heavier burden of hepatitis infection, poorer coagulation function, and more advanced tumour stage than patients without cirrhosis. Therefore, we concentrated on the prognoses of this subgroup of patients with HCC in the present study.

In the present study, univariate and multivariate analyses identified five risk factors for RFS and seven for OS in HCC patients with cirrhosis. Individual nomograms for RFS and OS were established based on these factors. The C-indices and calibration plots of the nomograms showed their promising accuracy and optimal consistency. The results of the internal validation also suggested the ideal performance of these prediction models. Comparison of the nomograms with four conventional staging systems revealed that our nomograms are superior to the staging systems in predicting the prognoses of HCC patients with cirrhosis. The results of this study further demonstrated that the nomograms were more suitable for predicting individual clinical events than commonly used staging systems [20].

Compared with routinely used staging systems, some new prognosis-related elements including AFP level, HBeAg, GGT level, MVI, satellite lesions, and histologic differentiation were integrated into the nomograms developed in the present study. Serum AFP level is the most common biomarker used for diagnosing HCC, and has been proven to have satisfactory sensitivity and specificity [30, 31]. Preoperative serum AFP level is normally used as an indicator of tumour burden and an predictor of the prognosis of HCC after hepatectomy [32, 33]. Serum HBeAg positivity indicates active viral replication and is associated with deterioration of HCC [34, 35]. A recent propensity score matching study showed that serum HBeAg positivity is an independent risk factor for RFS and OS in patients with HCC after curative surgery [36]. GGT could be abundantly produced by HCC cells and is a valuable biomarker for the diagnosis of HCC in patients with a low serum AFP level [37–39]. Higher serum GGT level is associated with larger tumour size, presence of vascular invasion, and advanced tumour stage [40, 41]. Similar to the present study, numerous studies have indicated that elevated GGT level is significantly associated with unfavourable prognosis after curative liver resection in patients with HCC [42, 43]. MVI is an established risk factor of early recurrence and poor survival after liver resection in patients with HCC [22, 44, 45]. The incidence of MVI is positively associated with tumour size and number [44]. In addition, the presence of satellite lesions indicate intrahepatic dissemination [23, 46, 47], which is a dangerous signal of early recurrence and short survival time after hepatectomy in patients with HCC [48, 49]. Poor histologic differentiation is considered an aggressive characteristic of HCC lesions [50]. Poor differentiation is associated with larger tumour size, upregulated AFP level, presence of MVI, and unfavourable prognosis in patients with HCC [51, 52].

This study has some limitations. First, as the prevalence of HBV infection in China is high, we only enrolled patients with a history of HBV infection. In addition, HBeAg positivity is one of the elements included in the nomograms. Therefore, the use of these models for predicting the prognoses of HCC of other aetiologies is limited. Second, this was a retrospective study. Thus, further prospective studies and external validations are necessary to corroborate the findings of this study. Third, the superiority of our nomograms compared to conventional staging systems should be cautiously interpreted. Multiple postoperative variables, which are helpful for postoperative decision-making, were incorporated in these nomograms. On the other hand, conventional staging systems are useful for guiding the treatment of all patients with HCC based on preoperative parameters.

## Conclusion

This study of a large cohort of patients with HCC demonstrated that there many clinicopathological differences between patients with HBV-associated HCC with or without cirrhosis. Univariate and multivariate analyses revealed that five clinical variables were significantly associated with RFS, whereas seven variables were significantly associated with OS in the study population. Using these variables, we constructed nomograms for predicting the RFS and OS of HCC patients with cirrhosis. These nomograms showed good accuracy and optimal performance when compared with conventional staging systems.

## Data Availability

The datasets generated and analysed during the current study are not publicly available due to patient privacy but are available from the corresponding author on reasonable request.

## Abbreviations

HCC: Hepatocellular carcinoma
HBV: Hepatitis B virus
HBV-DNA: Hepatitis B virus deoxyribonucleic acid
HBeAg: Hepatitis B e antigen
AFP: Alpha-fetoprotein
GGT: Gamma-glutamyl transpeptidase
MVI: Microvascular invasion
BCLC: Barcelona Clinic Liver Cancer
RFS: Recurrence-free survival
OS: Overall survival
